# Development and assessment of novel machine learning models to predict medication non-adherence risks in type 2 diabetics

**DOI:** 10.3389/fpubh.2022.1000622

**Published:** 2022-11-17

**Authors:** Mengting Li, Xiangyu Lu, HengBo Yang, Rong Yuan, Yong Yang, Rongsheng Tong, Xingwei Wu

**Affiliations:** ^1^Personalized Drug Therapy Key Laboratory of Sichuan Province, Department of Pharmacy, Sichuan Provincial People's Hospital, University of Electronic Science and Technology of China, Chengdu, China; ^2^Chinese Academy of Sciences Sichuan Translational Medicine Research Hospital, Chengdu, China; ^3^The Second Department of Hepatobiliary Surgery, Sichuan Academy of Medical Sciences and Sichuan Provincial People's Hospital, University of Electronic Science and Technology of China, Chengdu, China; ^4^School of Pharmacy, Chengdu Medical College, Chengdu, China; ^5^Endocrine Department, Sichuan Provincial People's Hospital, Chengdu, China

**Keywords:** medication adherence, T2D, machine learning, prediction model, ensemble model

## Abstract

**Background:**

Medication adherence is the main determinant of effective management of type 2 diabetes, yet there is no gold standard method available to screen patients with high-risk non-adherence. Developing machine learning models to predict high-risk non-adherence in patients with T2D could optimize management.

**Methods:**

This cross-sectional study was carried out on patients with T2D at the Sichuan Provincial People's Hospital from April 2018 to December 2019 who were examined for HbA1c on the day of the survey. Demographic and clinical characteristics were extracted from the questionnaire and electronic medical records. The sample was randomly divided into a training dataset and a test dataset with a radio of 8:2 after data preprocessing. Four imputing methods, five sampling methods, three screening methods, and 18 machine learning algorithms were used to groom data and develop and validate models. Bootstrapping was performed to generate the validation set for external validation and univariate analysis. Models were compared on the basis of predictive performance metrics. Finally, we validated the sample size on the best model.

**Results:**

This study included 980 patients with T2D, of whom 184 (18.8%) were defined as medication non-adherence. The results indicated that the model used modified random forest as the imputation method, random under sampler as the sampling method, Boruta as the feature screening method and the ensemble algorithms and had the best performance. The area under the receiver operating characteristic curve (AUC), F1 score, and area under the precision-recall curve (AUPRC) of the best model, among a total of 1,080 trained models, were 0.8369, 0.7912, and 0.9574, respectively. Age, present fasting blood glucose (FBG) values, present HbA1c values, present random blood glucose (RBG) values, and body mass index (BMI) were the most significant contributors associated with risks of medication adherence.

**Conclusion:**

We found that machine learning methods could be used to predict the risk of non-adherence in patients with T2D. The proposed model was well performed to identify patients with T2D with non-adherence and could help improve individualized T2D management.

## Introduction

Diabetes mellitus (DM) is a common chronic disease with disordered metabolism and hyperglycemia. Type 2 diabetes (T2D) accounts for more than 90% of diabetic cases (1, 2). As morbidity and prevalence continue to rise worldwide, T2D greatly increases healthcare costs and imposes a tremendous economic burden on society and public health systems (3, 4). Total healthcare costs for diabetics are estimated ~$2.1 trillion by 2030 (5).

Pharmacotherapy is one of the most commonly used treatment modalities for controlling the progression of chronic diseases, especially diabetes. In most cases, the benefits of high adherence to medications have been well-determined in diabetes (6–8). The extent to which patients follow prescribed treatments determines the outcome. However, poor adherence to oral hypoglycemic drugs is common in patients with T2D (9). As reported, between a third and a half of drugs prescribed for patients with T2D were not taken as recommended, and estimates varied widely depending on the population studied (10–13). Evidence suggested that non-adherence was an important contributor associated with poor glycemic control and other negative health outcomes, such as the increased risk of hospitalization and complications (14, 15). In a decade, studies indicated that telephone calls, text messages, and educational interventions played an important role in improving adherence to medication (16–18). However, for patients with good compliance, additional interventions are a waste of healthcare resources that are already limited. Thus, the early detection of patients with a high risk of poor adherence to medication is the premise of these effective interventions.

So, we considered whether it is possible to identify patients with a high risk of poor medication adherence early and provide individualized methods to improve their compliance. In our previous study, we reported predictive models of the risks of medication adherence in patients with T2D (19), and the area under the receiver operating characteristic curve (AUC) of the ensemble model was 0.866. The results confirmed that machine learning could be used to predict the risk of drug non-adherence in patients with T2D. Thus, in this study, we used a larger sample size, more variables, more data preprocessing algorithms, and machine learning algorithms to develop models that could more accurately predict medication adherence in patients with T2D.

## Methods

### Data sources and participants

The cross-sectional study was conducted at the Sichuan Provincial People's Hospital from 1 April 2018 to 31 December 2019. We performed a face-to-face questionnaire interview and filled out questionnaires according to the responses of the patients who participated in the survey. Participants were selected according to the following criteria: (1) diagnosed as patients with T2D; (2) examined HbA1c on the day of the questionnaire; (3) interested to take part in the survey and provide information to the investigators, as well as signed the informed consent forms; (4) received hypoglycemic agency treatment; and (5) over 18 years of age. Ethics approval was obtained through the Ethics Committee of the Sichuan Provincial People's Hospital (approval # 2018-53).

### Data collection and outcome definition

The data in this study were collected from electronic medical records (EMRs) and face-to-face questionnaires. Clinical laboratory results, such as HbA1c value and fasting blood glucose (FBG) value, were collected according to EMRs. Body mass index (BMI) was calculated using the following formula: BMI = weight (kg)/height^2^ (m^2^). Information on self-glycemic monitoring, diet, exercise, and mental state were provided by patients in face-to-face questionnaires. The questionnaire consists of four parts. The first part is about basic characteristics, including age, nationalities, waistline, occupation, marital status, and so on. The second part is related to self-glycemic monitoring, containing regular measurements frequency of FBG, measurement interval between previous and present, etc. The third part was about exercise, diet, and mental state. The last part was treatment regimen and medication adherence, in which we recorded the duration of the treatment regimen, type and dose of insulin used, etc. The adherence status, which was determined as the outcome variable, was defined according to the proportion of days covered (PDC). PDC higher than 80% was regarded as good medication compliance (20, 21).

### Data preprocessing

Data were preprocessed by removing (1) the variables with missing values >90%, (2) the variables with a single value occupying >90%, and (3) the variables with coefficients of variation < 0.01. After the above steps, the data were further processed.

### Data partition and dataset building

The data were randomly divided into two subsets (namely, training set and test set) at a ratio of 8:2, which would be used to train and test models, respectively.

Missing data were inevitable in practice. In case of questionable data or missing data in the part of the questionnaire, patients were contacted *via* telephone for certainty or addition. However, the clinical characteristics of the patients comprised several missing values, such as FBG and postprandial blood glucose (PBG). Missing data were filled in using four imputing methods, including not imputing (marked as Not), simple imputing, random forest, and modified random forest.

Due to the imbalanced data of medication adherence, five sampling methods were applied, including not sampling (marked as Not), Synthetic Minority Oversampling Technique (SMOTE), Borderline SMOTE, Random Over Sampler, and Random Under Sampler.

Three variable selection methods were considered in this study, including no screening (marked as Not), Boruta, and LassoCV. The importance of variables was evaluated according to the output of Boruta and LassoCV (variable importance scores). A high score suggested that the variable could improve predictive accuracy.

Thus, a total of 60 datasets were derived from the training set and set up by using four imputing methods, five sampling methods, and three feature screening methods.

### Model development

In this process, several machine learning algorithms were trained for binary classification and applied to develop predictive models, including AdaBoost, Extreme Gradient Boosting (XGBoost), gradient boosting, Bagging, Bernoulli Naive Bayes, Gaussian Naive Bayes, Multinomial Naive Bayes, decision tree, extra tree, K-nearest neighbor (KNN), linear discriminant analysis (LDA), quadratic discriminant analysis (QDA), logistic regression, passive-aggressive, random forest, Stochastic Gradient Descent (SGD), support vector machine (SVM), and ensemble algorithm. The ensemble algorithm summarized the output of the five best models [assessed by area under the receiver operating characteristic curve (AUC)] among the trained models and generated output according to the voting principle.

### Model evaluation

Internal validation was conducted with 10-fold cross-validation in 60 datasets, and 10 independent repeated values among indices were collected. Then, the test set was used for external validation. The predictive performances of those models were assessed by the AUC, accuracy, precision, recall, F1-score, and area under the precision-recall curve (AUPRC). AUPRC was calculated by taking the average of precision across all recall values corresponding to different thresholds, and a high value represented both high recall and precision (22, 23).

To elucidate the contribution of different imputing methods, sampling methods, screening methods, machine learning algorithms, and variables, univariate analysis was performed. The whole process could be described as follows: (1) before analysis, the test set was expanded using the Bootstrap method with 2,000 times resampling from the test set. (2) Additionally, the average performance metrics of each method were calculated, respectively. (3) Univariate analysis was used for statistical analysis. The highest values of performance metrics meant that the method was the best than others. If the average performance metrics of models when the variable was included were significantly higher than the average performance indicators when the variable was excluded (*P* < 0.05), the variable would be judged as a positive contribution to the prediction improvement.

**Figure 1:**
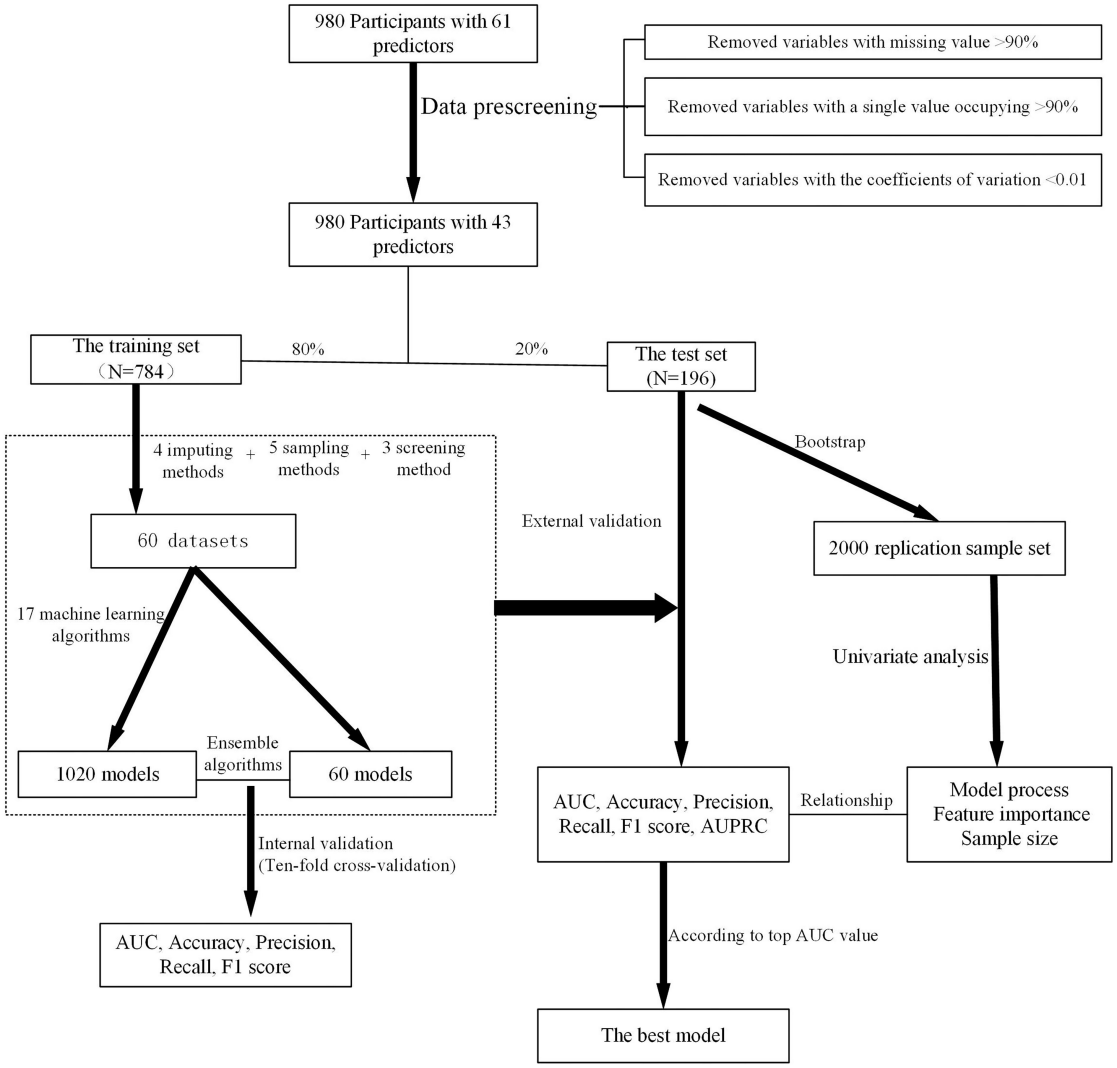
The schematic flow of the main steps in this study.

Above all, the overall process of model development and validation is shown in Figure 1.

### Sample size validation

The best model (assessed by AUC) was employed to estimate the impact of sample sizes on predictive performance (19). The total samples were randomly separated into 80% training set and 20% test set. First, 10% of the samples were randomly extracted from the training set to train the model, and AUC was evaluated in the test set. The training samples increased from 10 to 100% in increments of 10%. These steps were repeated 10 times so that ten independent repeated values of AUC were generated. The contribution of a sample size to improve the prediction performance of models was assessed according to the inflection point change of the line graph.

### Statistical analysis

Continuous variables were described by mean and standard deviation, whereas categorical variables were expressed in terms of frequencies and percentages. Analysis of variance (ANOVA) and rank sum test were used for univariate analysis.

Statistical analysis was implemented using the stats package, and model development was performed using the sklearn package in Python (*Python* Software Foundation, Python Language Reference, version 3.6.8) on PyCharm (developed by JetBrains.r.o., version 11.0.4). The results of variable valuation assessed using univariate analysis were summarized and presented by box plots using R (R software, version 4.0.2).

## Results

### Participant characteristics

Overall, 980 patients completed the survey, among which 571 were male and 409 were female. The mean age was 59.2 ± 11.9 years. In total, 184 patients were defined as having poor medication adherence (18.8%). Detailed characteristics of participants are shown in Table 1.

**Table 1:** The detailed information of participants.

**Variable**	**Identifier**	**Parameter**	**Value** ** (*N* = 980)**
**Basic characteristics**			
Age	X1	*N*	980
		Mean ± SD	59.2 ± 11.9
		Median	59
		Minimum, maximum	21, 90
Nationalities	X2	*N*	979
		Han	945 (96.5%)
		Tibetan	31 (3.2%)
		Qiang	3 (0.3%)
Gender	X3	*N*	980
		Male	571 (58.3%)
		Female	409 (41.7%)
Height (m)	X4	*N*	978
		Mean ± SD	1.6 ± 8.0
		Median	1.6
		Minimum, maximum	1.4, 1.9.0
Waistline (cm)	X5	*N*	913
		Mean ± SD	85.3 ± 9.5
		Median	83.3
		Minimum, maximum	66.6, 128.0
Weight (kg)	X6	*N*	976
		Mean ± SD	64.2 ± 10.5
		Median	64
		Minimum, maximum	40.0, 110.0
Marital status	X7	*N*	976
		Unmarried	9 (0.9%)
		Married	940 (96.3%)
		Divorced	4 (0.4%)
		Widowed	23 (2.4%)
Occupational status	X8	*N*	978
		Unemployed	133 (13.6%)
		Empolyed	358 (36.6%)
		Retirement	482 (49.3%)
		Others	5 (0.5%)
Education level	X9	*N*	978
		Illiteracy	92 (9.4%)
		Junior middle school	366 (37.4%)
		High school or special secondary school	264 (27.0%)
		College and above educational level	256 (26.2%)
Family history of diabetes mellitus	X10	*N*	970
		No	629 (64.8%)
		Yes	341 (35.2%)
BMI (kg/m^2^)	X11	*N*	975
		Mean ± SD	24.3 ± 3.3
		Median	24.0
		Minimum, maximum	16.2, 45.2
Health status scores (%)	X12	*N*	980
		Mean ± SD	77.3 ± 10.8
		Median	80
		Minimum, maximum	30, 100
**Clinical information**			
Course of diabetes (in months)	X13	*N*	980
		Mean ± SD	90.3 ± 76.5
		Median	72
		Minimum, maximum	1, 540
Medicare status	X14	*N*	518
		unreimbursement	233 (45.0%)
		reimbursement	285 (55.0%)
Frequency of FBG measurements	X15	*N*	980
		Irregular measurements	139 (14.2%)
		Two to three times a week	323 (33.0%)
		Three to four times a month	400 (40.8%)
		Two to three times per three months	118 (12.0%)
Interval of measurement (in days)	X16	*N*	613
		Mean ± SD	212.5 ± 213.7
		Median	150
		Minimum, maximum	2.0, 2920.0
Previous HbA_1c_ values (%)	X17	*N*	676
		≤ 7%	269 (39.8%)
		7%-9%	328 (48.5%)
		>9%	79 (11.7%)
Present HbA_1c_ values (%)	X18	*N*	980
		Mean ± SD	7.5 ± 1.6
		Median	7.1
		Minimum, maximum	4.6, 15.0
Present FBG level	X19	*N*	838
		3.8–6.1	54 (6.4%)
		6.1–7	257 (30.7%)
		≥7	527 (62.9%)
Present FBG values (mmoL/L)	X20	*N*	197
		Mean ± SD	9.3 ± 3.56
		Median	8.1
		Minimum, maximum	3.3, 22.0
Present RBG values (mmoL/L)	X21	*N*	517
		Mean ± SD	11.6 ± 5.1
		Median	10.4
		Minimum, maximum	3.1, 34.1
Present PBG values (mmoL/L)	X22	*N*	16
		Mean ± SD	9.8 ± 2.4
		Median	9.3
		Minimum, maximum	6.9, 13.8
Type of operation or other communicable diseases	X23	*N*	979
		No	775 (79.2%)
		Abdominal surgery	114 (11.6%)
		Thoracic surgery	31 (3.2%)
		Others	59 (6.0%)
Number of comorbid diseases	X24	*N*	979
		0	500 (51.1%)
		1	299 (30.5%)
		2	143 (14.6%)
		3	34 (3.5%)
		4	3 (0.3%)
Hypertension	X25	*N*	980
		No	663 (67.7%)
		Yes	317 (32.3%)
Hyperlipidemia	X26	*N*	979
		No	768 (78.4%)
		Yes	211 (21.6%)
With or without complications	X27	*N*	980
		No	884 (90.2%)
		Yes	96 (9.8%)
Vascular complications	X28	*N*	980
		No	977 (99.7%)
		Yes	3 (0.3%)
Neurological complication	X29	*N*	980
		No	926 (94.5%)
		Yes	54 (5.5%)
Complications with lesions of the extremities	X30	*N*	980
		No	975 (99.5%)
		Yes	5 (0.5%)
Ocular complications	X31	*N*	980
		No	973 (99.3%)
		Yes	7 (0.7%)
Nephropathy complications	X32	*N*	980
		No	972 (99.2%)
		Yes	8 (0.8%)
Complications(other diseases)	X33	*N*	980
		No	957 (97.7%)
		Yes	23 (2.3%)
**Exercise, diet and mental state**			
Intensity of exercise	X34	*N*	980
		None	153 (15.6%)
		Low intensity	664 (67.8%)
		Moderate intensity	124 (12.7%)
		High intensity	39 (3.9%)
Exercise session (mins/day)	X35	*N*	980
		Mean ± SD	53.4 ± 55.4
		Median	45
		Minimum, maximum	0, 600
Had a ration and reasonable eating	X36	*N*	980
		No	256 (26.1%)
		Yes	724 (73.9%)
Sleep duration	X37	*N*	980
		Good	453 (46.2%)
		Ordinary	333 (34.0%)
		Lose sleep	194 (19.8%)
Psychological status	X38	*N*	980
		Well	459 (46.8%)
		General	493 (50.3%)
		Depression	28 (2.9%)
EQ-5D scores	X39	*N*	980
		Mean ± SD	0.9 ± 0.1
		Median	1
		Minimum, maximum	0.5, 1.0
**Treatment regimen and medication adherence**		
Compliance	X40	N	980
		No	183 (18.6%)
		Yes	797 (83.4%)
Duration of treatment regimen (in months)	X41	N	979
		Mean ± SD	24.8 ± 34.0
		Median	12
		Minimum, maximum	1.0, 240.0
Type of insulin used	X42	N	980
		0	731 (74.6%)
		1	228 (23.3%)
		2	21 (2.1%)
Use of insulin	X43	N	980
		No	744 (75.9%)
		Yes	236 (24.1%)
Times of insulin use	X44	N	980
		0	730 (74.5%)
		1	104 (10.6%)
		2	112 (11.4%)
		3	15 (1.5%)
		4	19 (2.0%)
Dose of basal insulin (U)	X45	N	980
		Mean ± SD	2.0 ± 5.7
		Median	0
		Minimum, maximum	0, 35
Dose of non-basal insulin in morning (U)	X46	N	980
		Mean ± SD	2.2 ± 5.8
		Median	0
		Minimum, maximum	0, 33
Dose of non-basal insulin in noon (U)	X47	N	980
		Mean ± SD	0.4 ± 2.5
		Median	0
		Minimum, maximum	0, 32
Dose of non-basal insulin in afternoon (U)	X48	N	980
		Mean ± SD	2.2 ± 5.7
		Median	0
		Minimum, maximum	0, 32
Number of oral drugs	X49	N	980
		0	71 (7.2%)
		1	328 (33.5%)
		2	419 (42.8%)
		3	153 (15.6%)
		4	8 (0.8%)
		5	1 (0.1%)
Use of other types of drugs	X50	N	979
		None	804 (82.1%)
		National medicine	11 (1.1%)
		Chinese medicine	88 (9.0%)
		Health care products	71 (7.3%)
		Others	5 (0.5%)
Use of metformin	X51	N	979
		None	313 (32.0%)
		Once a day	175 (17.9%)
		Twice a day	399 (40.8%)
		Three times a day	92 (9.3%)
Dose of metformin	X52	N	976
		None	313 (32.1%)
		0.25 g	50 (5.1%)
		0.425 g	2 (0.2%)
		0.5 g	154 (15.8%)
		0.75 g	1 (0.1%)
		0.85 g	447 (45.8%)
		1.0 g	9 (0.9%)
Type of manufacturers of metformin	X53	N	976
		Unknown	313 (32.1%)
		Generic drugs	205 (21.0%)
		Guthentic drugs	458 (46.9%)
α-Glucosidase inhibitors	X54	N	980
		No	616 (62.9%)
		Yes	364 (37.1%)
Sulfonylureas	X55	N	980
		No	637 (65.0%)
		Yes	343 (35.0%)
Glinides	X56	*N*	980
		No	911 (93.0%)
		Yes	69 (7.0%)
DPP-4 inhibitors	X57	*N*	980
		No	845 (86.2%)
		Yes	135 (13.8%)
Thiazolidinediones	X58	*N*	980
		No	928 (94.7%)
		Yes	52 (5.3%)
GLP-1 RAs	X59	*N*	980
		No	979 (99.9%)
		Yes	1 (0.1%)
SGLT2 inhibitors	X60	*N*	980
		No	976 (99.6%)
		Yes	4 (0.4%)
Use of Chinese medicine	X61	*N*	980
		No	974 (99.4%)
		Yes	6 (0.6%)

BMI, body mass index; HbA1c, glycated hemoglobin; FBG, fasting blood glucose; RBG, random blood glucose; EQ-5D, EuroQol five dimensions questionnaire; DPP-4 inhibitors, dipeptidylpeptidase-4 inhibitors; GLP-1 Ras, glucagon-like peptide-1 receptor agonists; SGLT2 inhibitors, sodium-dependent glucose transporters 2 inhibitors.

### Dataset building

After data preprocessing, 43 variables were retained, and 18 variables were deleted. Sixty datasets were set up by applying different imputing methods, sampling methods, and screening methods with 43 variables. Additionally, the different number of variables and samples in each dataset is listed in Table 2.

**Table 2:** The detailed information of 60 datasets.

**Number**	**Imputing methods**	**Sampling methods**	**Screening methods**	**Number of variables**	**Number of train samples**
1	Not	Not	Not	33	773
2	Not	Not	Boruta	21	773
3	Not	Not	LassoCV	12	773
4	Not	SMOTE	Not	33	1,260
5	Not	SMOTE	Boruta	16	1,260
6	Not	SMOTE	LassoCV	22	1,260
7	Not	Borderline SMOTE	Not	33	1,260
8	Not	Borderline SMOTE	Boruta	17	1,260
9	Not	Borderline SMOTE	LassoCV	23	1,260
10	Not	Random OverSampler	Not	33	1,260
11	Not	Random OverSampler	Boruta	16	1,260
12	Not	Random OverSampler	LassoCV	20	1,260
13	Not	Random UnderSampler	Not	33	286
14	Not	Random UnderSampler	Boruta	21	286
15	Not	Random UnderSampler	LassoCV	8	286
16	Simple	Not	Not	43	784
17	Simple	Not	Boruta	21	784
18	Simple	Not	LassoCV	11	784
19	Simple	SMOTE	Not	43	1,274
20	Simple	SMOTE	Boruta	21	1,274
21	Simple	SMOTE	LassoCV	30	1,274
22	Simple	Borderline SMOTE	Not	43	1,274
23	Simple	Borderline SMOTE	Boruta	20	1,274
24	Simple	Borderline SMOTE	LassoCV	30	1,274
25	Simple	Random OverSampler	Not	43	1,274
26	Simple	Random OverSampler	Boruta	19	1,274
27	Simple	Random OverSampler	LassoCV	25	1,274
28	Simple	Random UnderSampler	Not	43	294
29	Simple	Random UnderSampler	Boruta	26	294
30	Simple	Random UnderSampler	LassoCV	9	294
31	Random forest	Not	Not	43	784
32	Random forest	Not	Boruta	23	784
33	Random forest	Not	LassoCV	12	784
34	Random forest	SMOTE	Not	43	1,274
35	Random forest	SMOTE	Boruta	22	1,274
36	Random forest	SMOTE	LassoCV	30	1,274
37	Random forest	Borderline SMOTE	Not	43	1,274
38	Random forest	Borderline SMOTE	Boruta	21	1,274
39	Random forest	Borderline SMOTE	LassoCV	31	1,274
40	Random forest	Random OverSampler	Not	43	1,274
41	Random forest	Random OverSampler	Boruta	18	1,274
42	Random forest	Random OverSampler	LassoCV	25	1,274
43	Random forest	Random UnderSampler	Not	43	294
44	Random forest	Random UnderSampler	Boruta	24	294
45	Random forest	Random UnderSampler	LassoCV	19	294
46	Modified random forest	Not	Not	43	784
47	Modified random forest	Not	Boruta	22	784
48	Modified random forest	Not	LassoCV	13	784
49	Modified random forest	SMOTE	Not	43	1,274
50	Modified Random Forest	SMOTE	Boruta	21	1,274
51	Modified Random Forest	SMOTE	LassoCV	31	1,274
52	Modified Random Forest	Borderline SMOTE	Not	43	1,274
53	Modified Random Forest	Borderline SMOTE	Boruta	22	1,274
54	Modified Random Forest	Borderline SMOTE	LassoCV	30	1,274
55	Modified Random Forest	Random OverSampler	Not	43	1,274
56	Modified Random Forest	Random OverSampler	Boruta	18	1,274
57	Modified Random Forest	Random OverSampler	LassoCV	24	1,274
58	Modified Random Forest	Random UnderSampler	Not	43	294
59	Modified Random Forest	Random UnderSampler	Boruta	24	294
60	Modified Random Forest	Random UnderSampler	LassoCV	18	294

### Model validation

**Figure 2:**
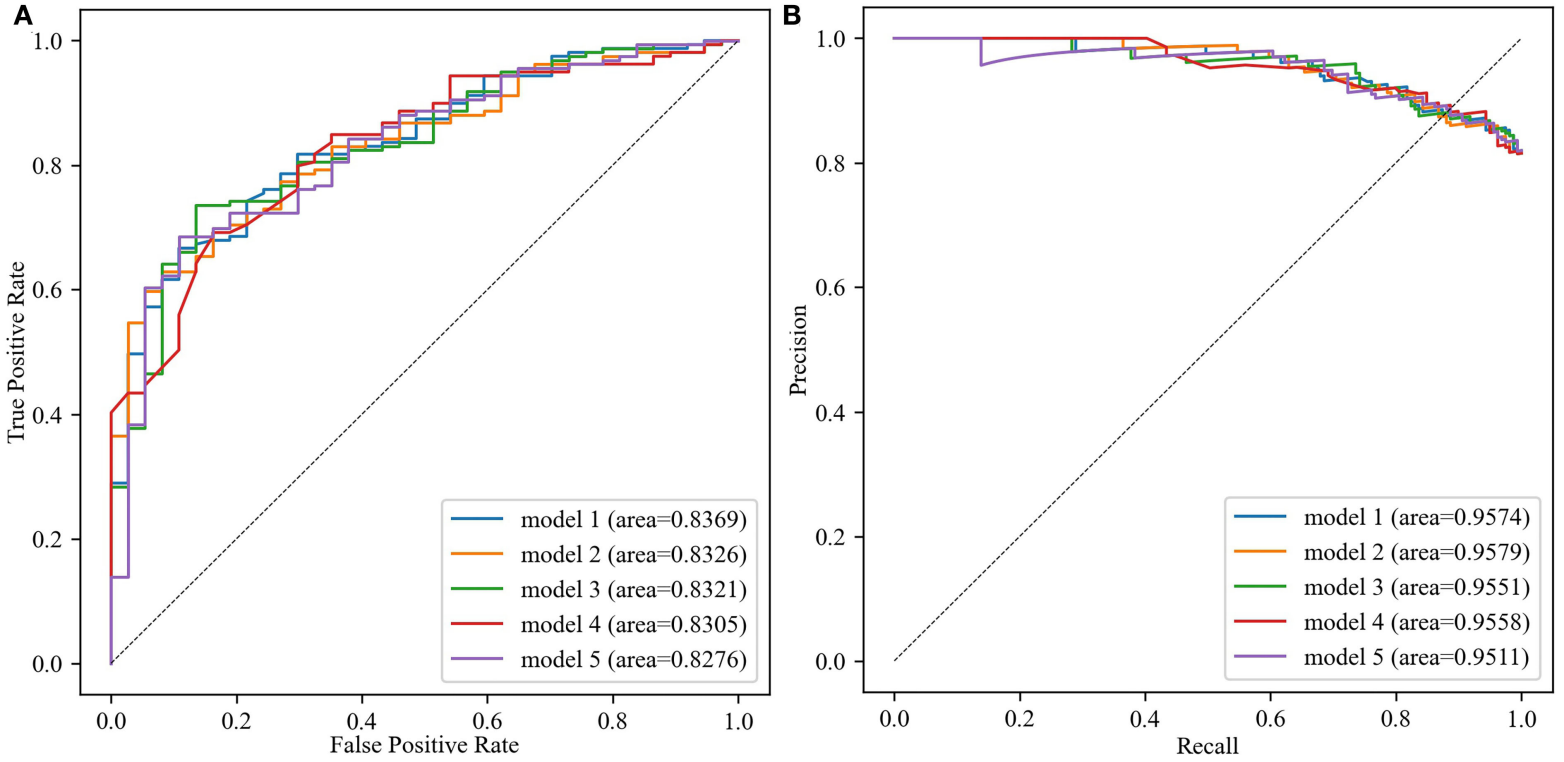
The area under the receiver operating characteristic curve (AUC) and area under the precision-recall curve (AUPRC) of the best five models. **(A)** The receiver operator characteristic curve. **(B)** The precision-recall curves.

A total of 1,080 models were validated in the test set, considered as external validation, and the performance metrics were output. As shown in Table 3, the best five models were listed in sequence according to the AUC value. The best model (model 1) was applied the ensemble algorithm and trained in the No. 59 dataset (applied modified random forest as imputing method, random under sampler as sampling method, and Boruta as screening method). AUC, accuracy, precision, recall, F1 score, and AUPRC of the best model (model 1) were 0.8369, 0.9474, 0.6792, 0.7912, and 0.9574, respectively (Table 3; Figure 2). Especially in unbalanced data, the high value of AUPRC indicated that the best model (model 1) performed well to identify patients at risk for non-adherence.

**Table 3:** The summary of the performance of five best models.

**ID**	**Algorithms**	**AUC**	**Accuracy**	**Precision**	**Recall**	**F1 Score**	**AUPRC**
Model 1	Ensemble	0.8369	0.7092	0.9474	0.6792	0.7912	0.9574
Model 2	Ensemble	0.8326	0.7041	0.9469	0.6730	0.7868	0.9579
Model 3	Bernoulli Naive Bayes	0.8321	0.7500	0.9435	0.7358	0.8269	0.9551
Model 4	Ensemble	0.8305	0.8010	0.9000	0.8491	0.8738	0.9558
Model 5	SGD	0.8276	0.6786	0.9615	0.6289	0.7605	0.9511

AUC, area under the receiver operating characteristic curve; AUPRC, area under the precision-recall curve.

As shown in Table 4, the effects of various factors on model performance were compared using univariate analysis. With a decrease in the number of samples (AUC=-0.071, *P* < 0.0001) and an increase in the number of variables (AUC=0.047, *P* < 0.0001), the prediction model would achieve a high AUC value. Among the three imputing methods, modified random forest (AUC = 0.726 ± 0.076, vs. not 0.657 ± 0.075, simple 0.702 ± 0.087, and random forest 0.723 ± 0.081, *P* < 0.0001) was performed to improve performance of models, as well as random under sampler (AUC = 0.724 ± 0.076, vs. not 0.723 ± 0.080, random over sampler 0.698 ± 0.090, SMOTE 0.683 ± 0.086, and Border line SMOTE 0.682 ± 0.081, *P* < 0.0001) in five sampling methods, and Boruna (AUC = 0.709 ± 0.083, vs. not 0.700 ± 0.084, and LassoCV 0.698 ± 0.087, *P* < 0.0001) in three screening methods. In addition, the ensemble algorithm also performed well compared with other 17 algorithms (AUC = 0.790 ± 0.053, *P* < 0.0001). It should be mentioned that the above results were the same as the methods applied in the best model (model 1).

**Table 4:** The results of univariate analysis ($\bar{x}$ ± SD).

**Classification**	**AUC**	**Accuracy**	**Precision**	**Recall**	**F1 score**
**Number of samples**	–0.071	0.251	–0.134	0.236	0.255
	*P* < 0.0001	*P* < 0.0001	*P* < 0.0001	*P* < 0.0001	*P* < 0.0001
**Number of variables**	0.047	0.063	0.024	0.040	0.056
	*P* < 0.0001	*P* < 0.0001	*P* < 0.0001	*P* < 0.0001	*P* < 0.0001
**Imputing methods**					
Not	0.657 ± 0.075	0.701 ± 0.088	0.859 ± 0.039	0.762 ± 0.143	0.799 ± 0.078
Simple	0.702 ± 0.087	0.723 ± 0.094	0.863 ± 0.047	0.791 ± 0.157	0.813 ± 0.087
Random Forest	0.723 ± 0.081	0.733 ± 0.079	0.871 ± 0.046	0.795 ± 0.136	0.822 ± 0.070
Modified Random Forest	0.726 ± 0.076	0.735 ± 0.079	0.871 ± 0.045	0.797 ± 0.136	0.824 ± 0.070
P values	*P* < 0.0001	*P* < 0.0001	*P* < 0.0001	*P* < 0.0001	*P* < 0.0001
**Sampling methods**					
Not	0.723 ± 0.080	0.802 ± 0.039	0.832 ± 0.035	0.951 ± 0.062	0.885 ± 0.028
Random over sampler	0.698 ± 0.090	0.711 ± 0.071	0.873 ± 0.041	0.757 ± 0.112	0.805 ± 0.062
Random under sampler	0.724 ± 0.076	0.623 ± 0.068	0.907 ± 0.042	0.598 ± 0.086	0.716 ± 0.068
SMOTE	0.683 ± 0.086	0.741 ± 0.068	0.859 ± 0.033	0.815 ± 0.089	0.834 ± 0.052
Borderline SMOTE	0.682 ± 0.081	0.738 ± 0.064	0.859 ± 0.032	0.811 ± 0.084	0.832 ± 0.050
*P* values	*P* < 0.0001	*P* < 0.0001	*P* < 0.0001	*P* < 0.0001	*P* < 0.0001
**Screening methods**					
Not	0.700 ± 0.084	0.722 ± 0.091	0.865 ± 0.044	0.786 ± 0.151	0.813 ± 0.082
Lasso	0.698 ± 0.087	0.724 ± 0.086	0.865 ± 0.044	0.789 ± 0.144	0.816 ± 0.077
Boruta	0.709 ± 0.083	0.722 ± 0.080	0.868 ± 0.045	0.783 ± 0.136	0.814 ± 0.073
*P* values	*P* < 0.0001	*P* < 0.0001	*P* < 0.0001	*P* < 0.0001	*P* < 0.0001
**Algorithms**					
Logistic regression	0.716 ± 0.064	0.732 ± 0.068	0.869 ± 0.043	0.797 ± 0.127	0.823 ± 0.060
SGD	0.693 ± 0.095	0.727 ± 0.078	0.874 ± 0.054	0.788 ± 0.150	0.816 ± 0.075
KNN	0.667 ± 0.085	0.711 ± 0.073	0.854 ± 0.043	0.784 ± 0.135	0.809 ± 0.063
Decision tree	0.672 ± 0.065	0.682 ± 0.106	0.870 ± 0.051	0.726 ± 0.182	0.774 ± 0.110
Gaussian Naive Bayes	0.673 ± 0.086	0.689 ± 0.075	0.874 ± 0.038	0.722 ± 0.106	0.786 ± 0.064
Bernoulli Naive Bayes	0.753 ± 0.069	0.731 ± 0.060	0.881 ± 0.041	0.777 ± 0.099	0.821 ± 0.051
Multinomial Naive Bayes	0.661 ± 0.084	0.678 ± 0.088	0.853 ± 0.043	0.736 ± 0.157	0.779 ± 0.080
SVM	0.698 ± 0.057	0.752 ± 0.070	0.850 ± 0.042	0.849 ± 0.127	0.842 ± 0.061
QDA	0.689 ± 0.091	0.727 ± 0.069	0.869 ± 0.041	0.786 ± 0.109	0.819 ± 0.058
Random forest	0.743 ± 0.057	0.769 ± 0.093	0.862 ± 0.043	0.861 ± 0.159	0.850 ± 0.084
Extra tree	0.624 ± 0.080	0.679 ± 0.090	0.853 ± 0.045	0.739 ± 0.157	0.780 ± 0.087
LDA	0.735 ± 0.070	0.738 ± 0.063	0.880 ± 0.040	0.789 ± 0.109	0.826 ± 0.054
Passive aggressive	0.620 ± 0.090	0.657 ± 0.073	0.854 ± 0.042	0.699 ± 0.105	0.764 ± 0.066
AdaBoost	0.736 ± 0.061	0.725 ± 0.078	0.873 ± 0.044	0.782 ± 0.138	0.815 ± 0.071
Bagging	0.724 ± 0.059	0.746 ± 0.099	0.860 ± 0.038	0.827 ± 0.158	0.832 ± 0.091
Gradient boosting	0.730 ± 0.056	0.738 ± 0.084	0.866 ± 0.042	0.808 ± 0.145	0.826 ± 0.075
XGBoost	0.717 ± 0.068	0.756 ± 0.081	0.859 ± 0.039	0.842 ± 0.136	0.842 ± 0.073
Ensemble	0.790 ± 0.053	0.776 ± 0.067	0.886 ± 0.045	0.838 ± 0.122	0.854 ± 0.058
*P* values	*P* < 0.0001	*P* < 0.0001	*P* < 0.0001	*P* < 0.0001	*P* < 0.0001

### Feature selection and validation

**Figure 3:**
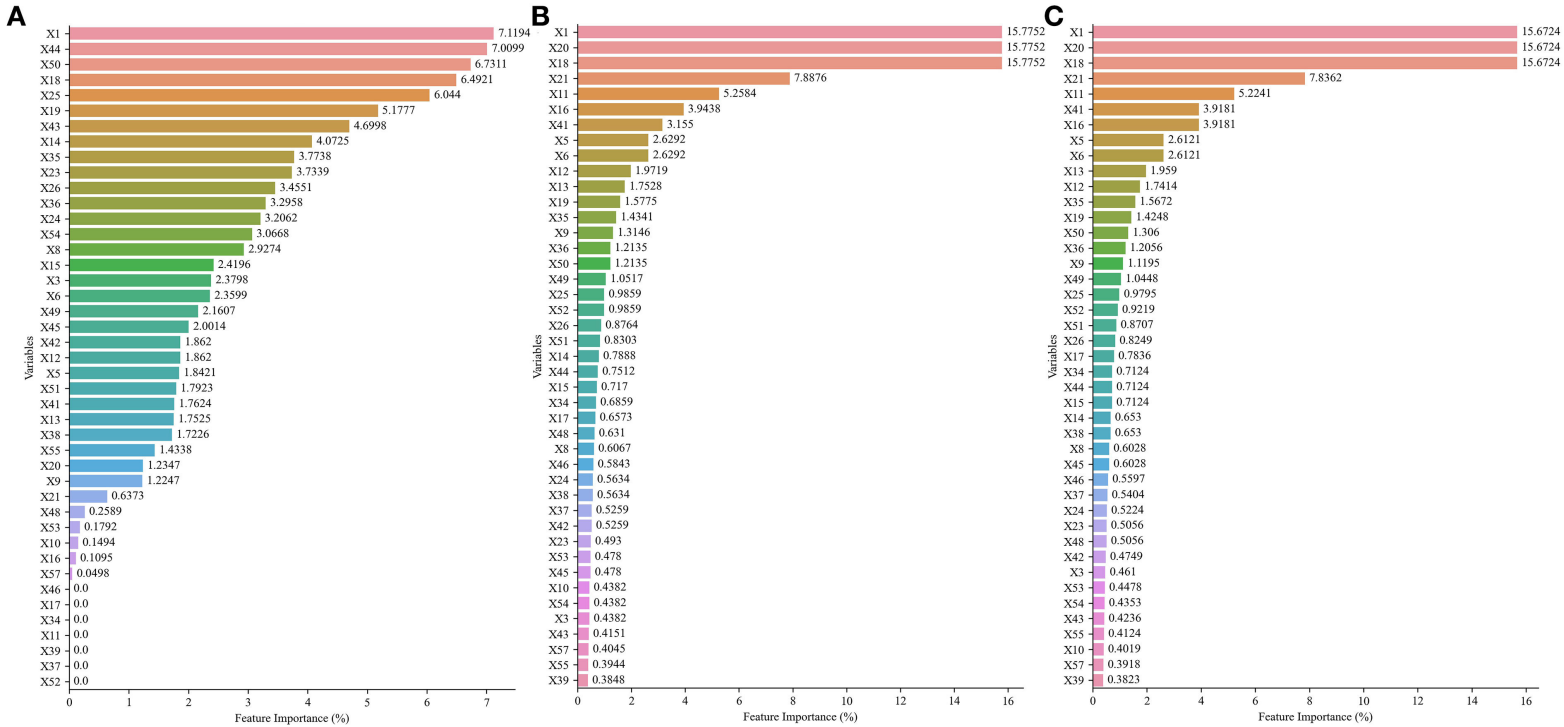
The importance scores and ranking of each variable in No. 27 dataset, No. 44 dataset, and No. 59 dataset with different variable selection methods. **(A)** Details of No. 27 dataset. **(B)** Details of No. 44 dataset. **(C)** Details of No. 59 dataset.

The best five models involved the following three datasets: No. 27, No. 44, and No. 59. In those datasets, the variable importance scores are ranked in Figure 3. Age, times of insulin use, use of other types of drugs, present HbA1c values, and hypertension were top 5 highest variable importance in No. 27 dataset (Figure 3A). The top 5 variables with the highest importance score in No. 44 dataset and No. 59 dataset were age, present FBG values, present HbA1c values, present random blood glucose (RBG) values, and BMI (Figures 3B,C).

**Figure 4:**
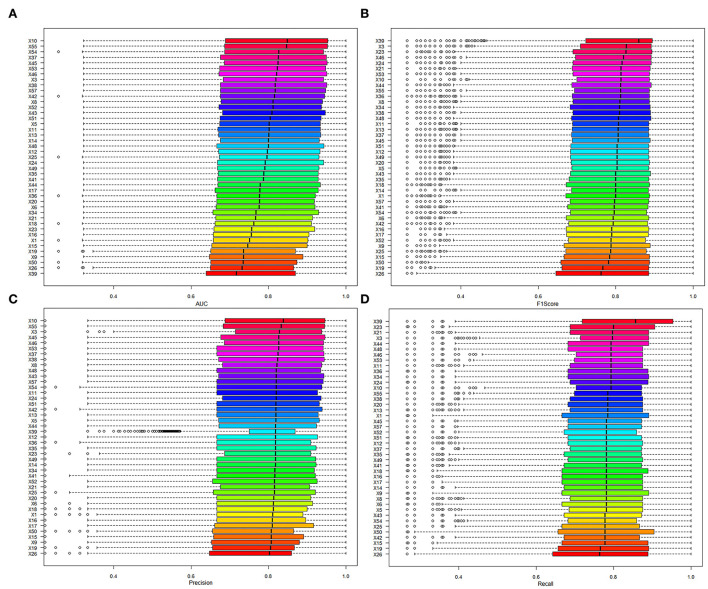
The model performance of models when the variables were included. **(A)** The results of AUC. **(B)** The results of the F1 score. **(C)** The results of precision. **(D)** The results of recall.

In addition, the contribution of variables was evaluated by comparing the AUC of models to identify whether the variable was included or excluded. In addition, the mean AUC of variables was from 0.689 to 0.724 in the included cohort and between 0.669 and 0.762 in the excluded cohort (details in Table 5; Figure 4). The variable that had higher AUC when the variable was included would be considered as a positive contribution to the prediction model. Those variables provided positive contributions and were in line with variables that had high variable importance scores, which was output in No. 59 dataset (the best model applied).

**Table 5:** The influence of model performances whether the variable was included or excluded.

**Variables**	**Code of**	**Included or**	**AUC**	**Accuracy**	**Precision**	**Recall**	**F1 Score**
	**variables**	**excluded**	**Mean ±SD**	**Mean ±SD**	**Mean ±SD**	**Mean ±SD**	**Mean ±SD**
Age	X1	No	0.672 ± 0.085	0.671-0.672	0.733 ± 0.070	0.732-0.733	0.859 ± 0.032
		Yes	0.707 ± 0.084	0.707-0.707	0.721 ± 0.088	0.721-0.721	0.867 ± 0.046
			*P* < 0.0001	*P* < 0.0001	*P* < 0.0001	*P* < 0.0001	*P* < 0.0001
Gender	X3	No	0.712 ± 0.128	0.698 ± 0.122	0.717 ± 0.142	0.716 ± 0.180	0.709 ± 0.148
		Yes	0.807 ± 0.142	0.780 ± 0.116	0.815 ± 0.142	0.791 ± 0.135	0.795 ± 0.118
			*P* < 0.0001	*P* < 0.0001	*P* < 0.0001	*P* < 0.0001	*P* < 0.0001
Waistline (cm)	X5	No	0.760 ± 0.141	0.747 ± 0.122	0.775 ± 0.145	0.773 ± 0.167	0.767 ± 0.140
		Yes	0.797 ± 0.145	0.766 ± 0.122	0.800 ± 0.150	0.771 ± 0.141	0.778 ± 0.127
			*P* < 0.0001	*P* < 0.0001	*P* < 0.0001	*P* = 0.0478	*P* = 0.0117
Weight (Kg)	X6	No	0.780 ± 0.142	0.765 ± 0.115	0.796 ± 0.140	0.796 ± 0.150	0.788 ± 0.125
		Yes	0.784 ± 0.146	0.758 ± 0.125	0.789 ± 0.151	0.765 ± 0.151	0.769 ± 0.133
			*P* = 0.2130	*P* = 0.0284	*P* = 0.2102	*P* < 0.0001	*P* < 0.0001
Occupational status	X8	No	0.688 ± 0.102	0.701 ± 0.110	0.718 ± 0.124	0.755 ± 0.202	0.729 ± 0.154
		Yes	0.802 ± 0.145	0.771 ± 0.121	0.805 ± 0.149	0.776 ± 0.139	0.783 ± 0.125
			*P* < 0.0001	*P* < 0.0001	*P* < 0.0001	*P* = 0.1838	*P* < 0.0001
Education level	X9	No	0.815 ± 0.138	0.776 ± 0.117	0.810 ± 0.145	0.778 ± 0.135	0.786 ± 0.120
		Yes	0.759 ± 0.146	0.747 ± 0.125	0.776 ± 0.149	0.768 ± 0.162	0.764 ± 0.139
			*P* < 0.0001	*P* < 0.0001	*P* < 0.0001	*P* = 0.0686	*P* < 0.0001
Family history of diabetes mellitus	X10	No	0.760 ± 0.141	0.747 ± 0.121	0.775 ± 0.145	0.769 ± 0.162	0.765 ± 0.138
		Yes	0.819 ± 0.144	0.777 ± 0.122	0.814 ± 0.151	0.778 ± 0.133	0.787 ± 0.121
			*P* < 0.0001	*P* < 0.0001	*P* < 0.0001	*P* = 0.0558	*P* < 0.0001
BMI (kg/m^2^)	X11	No	0.767 ± 0.141	0.750 ± 0.122	0.778 ± 0.146	0.768 ± 0.163	0.766 ± 0.138
		Yes	0.795 ± 0.147	0.766 ± 0.122	0.800 ± 0.150	0.775 ± 0.142	0.779 ± 0.126
			*P* < 0.0001	*P* < 0.0001	*P* < 0.0001	*P* < 0.0001	*P* = 0.0002
Health status scores (%)	X12	No	0.733 ± 0.130	0.736 ± 0.116	0.761 ± 0.135	0.777 ± 0.183	0.761 ± 0.146
		Yes	0.795 ± 0.146	0.764 ± 0.123	0.797 ± 0.150	0.771 ± 0.143	0.777 ± 0.128
			*P* < 0.0001	*P* < 0.0001	*P* < 0.0001	*P* = 0.0001	*P* = 0.0136
Course of diabetes (in months)	X13	No	0.736 ± 0.128	0.735 ± 0.117	0.761 ± 0.136	0.766 ± 0.182	0.756 ± 0.146
		Yes	0.795 ± 0.146	0.765 ± 0.123	0.798 ± 0.150	0.774 ± 0.143	0.778 ± 0.127
			*P* < 0.0001	*P* < 0.0001	*P* < 0.0001	*P* = 0.9355	*P* < 0.0001
Medicare status	X14	No	0.771 ± 0.146	0.755 ± 0.123	0.785 ± 0.147	0.774 ± 0.158	0.772 ± 0.136
		Yes	0.796 ± 0.143	0.763 ± 0.122	0.796 ± 0.149	0.771 ± 0.144	0.775 ± 0.127
			*P* < 0.0001	*P* = 0.0008	*P* < 0.0001	*P* = 0.0906	*P* = 0.9577
Frequency of FBG measurements	X15	No	0.810 ± 0.140	0.776 ± 0.116	0.810 ± 0.144	0.785 ± 0.137	0.790 ± 0.119
		Yes	0.768 ± 0.145	0.750 ± 0.125	0.779 ± 0.150	0.765 ± 0.159	0.765 ± 0.137
			*P* < 0.0001	*P* < 0.0001	*P* < 0.0001	*P* < 0.0001	*P* < 0.0001
Interval of measurement (in days)	X16	No	0.794 ± 0.143	0.766 ± 0.121	0.798 ± 0.148	0.776 ± 0.148	0.779 ± 0.129
		Yes	0.772 ± 0.146	0.752 ± 0.123	0.783 ± 0.149	0.768 ± 0.155	0.768 ± 0.134
			*P* < 0.0001	*P* < 0.0001	*P* < 0.0001	*P* = 0.0736	*P* < 0.0001
Previous HbA1c value (%)	X17	No	0.783 ± 0.143	0.762 ± 0.120	0.793 ± 0.145	0.776 ± 0.151	0.777 ± 0.131
		Yes	0.785 ± 0.148	0.755 ± 0.125	0.787 ± 0.153	0.767 ± 0.151	0.768 ± 0.132
			*P* = 0.5126	*P* = 0.0201	*P* = 0.0623	*P* = 0.0117	*P* = 0.0009
Present HbA1c values (%)	X18	No	0.881 ± 0.116	0.805 ± 0.115	0.849 ± 0.150	0.787 ± 0.086	0.808 ± 0.095
		Yes	0.776 ± 0.144	0.756 ± 0.122	0.787 ± 0.147	0.771 ± 0.155	0.771 ± 0.134
			*P* < 0.0001	*P* < 0.0001	*P* < 0.0001	*P* = 0.2544	*P* < 0.0001
Present FBG level	X19	No	0.812 ± 0.142	0.777 ± 0.118	0.812 ± 0.146	0.786 ± 0.135	0.791 ± 0.120
		Yes	0.757 ± 0.142	0.742 ± 0.124	0.771 ± 0.148	0.760 ± 0.164	0.758 ± 0.140
			*P* < 0.0001	*P* < 0.0001	*P* < 0.0001	*P* < 0.0001	*P* < 0.0001
Present FBG values (mmoL/L)	X20	No	0.781 ± 0.146	0.757 ± 0.125	0.787 ± 0.150	0.767 ± 0.159	0.769 ± 0.137
		Yes	0.784 ± 0.144	0.761 ± 0.121	0.792 ± 0.147	0.775 ± 0.147	0.776 ± 0.129
			*P* < 0.0001	*P* < 0.0001	*P* < 0.0001	*P* < 0.0001	*P* < 0.0001
Present RBG values (mmoL/L)	X21	No	0.678 ± 0.083	0.678-0.678	0.708 ± 0.089	0.708-0.708	0.864 ± 0.042
		Yes	0.719 ± 0.082	0.719-0.719	0.734 ± 0.083	0.733-0.734	0.868 ± 0.046
			*P* < 0.0001	*P* < 0.0001	*P* < 0.0001	*P* < 0.0001	*P* < 0.0001
Type of operation or other communicable diseases	X23	No	0.803 ± 0.141	0.742 ± 0.131	0.768 ± 0.161	0.725 ± 0.132	0.739 ± 0.131
		Yes	0.777 ± 0.146	0.765 ± 0.119	0.798 ± 0.143	0.788 ± 0.154	0.785 ± 0.130
			*P* < 0.0001	*P* < 0.0001	*P* < 0.0001	*P* < 0.0001	*P* < 0.0001
Number of comorbid diseases	X24	No	0.767 ± 0.139	0.744 ± 0.121	0.771 ± 0.146	0.760 ± 0.159	0.759 ± 0.136
		Yes	0.794 ± 0.148	0.769 ± 0.122	0.804 ± 0.149	0.780 ± 0.145	0.784 ± 0.128
			*P* < 0.0001	*P* < 0.0001	*P* < 0.0001	*P* < 0.0001	*P* < 0.0001
Hypertension	X25	No	0.727 ± 0.130	0.776 ± 0.094	0.808 ± 0.110	0.850 ± 0.140	0.824 ± 0.109
		Yes	0.793 ± 0.145	0.756 ± 0.127	0.788 ± 0.154	0.758 ± 0.149	0.765 ± 0.133
			*P* < 0.0001	*P* < 0.0001	*P* < 0.0001	*P* < 0.0001	*P* < 0.0001
Hyperlipidemia	X26	No	0.827 ± 0.138	0.793 ± 0.108	0.830 ± 0.137	0.801 ± 0.109	0.808 ± 0.100
		Yes	0.754 ± 0.142	0.737 ± 0.126	0.764 ± 0.150	0.753 ± 0.171	0.751 ± 0.145
			*P* < 0.0001	*P* < 0.0001	*P* < 0.0001	*P* < 0.0001	*P* < 0.0001
Intensity of exercise	X34	No	0.787 ± 0.139	0.759 ± 0.120	0.789 ± 0.145	0.768 ± 0.147	0.772 ± 0.129
		Yes	0.780 ± 0.150	0.759 ± 0.125	0.792 ± 0.151	0.776 ± 0.155	0.776 ± 0.134
			*P* = 0.0188	*P* = 0.4927	*P* = 0.1874	*P* = 0.0002	*P* = 0.0145
Exercise session (mins/day)	X35	No	0.751 ± 0.139	0.745 ± 0.118	0.774 ± 0.142	0.784 ± 0.174	0.770 ± 0.141
		Yes	0.791 ± 0.145	0.763 ± 0.123	0.795 ± 0.150	0.769 ± 0.145	0.775 ± 0.129
			*P* < 0.0001	*P* < 0.0001	*P* < 0.0001	*P* < 0.0001	*P* = 0.8408
Had a ration and reasonable eating	X36	No	0.769 ± 0.141	0.722 ± 0.139	0.748 ± 0.164	0.687 ± 0.154	0.710 ± 0.150
		Yes	0.784 ± 0.145	0.762 ± 0.121	0.794 ± 0.147	0.778 ± 0.149	0.778 ± 0.129
			*P* = 0.0545	*P* < 0.0001	*P* < 0.0001	*P* < 0.0001	*P* < 0.0001
Sleep duration	X37	No	0.758 ± 0.137	0.749 ± 0.117	0.777 ± 0.140	0.776 ± 0.161	0.770 ± 0.135
		Yes	0.807 ± 0.148	0.768 ± 0.126	0.803 ± 0.155	0.769 ± 0.141	0.778 ± 0.129
			*P* < 0.0001	*P* < 0.0001	*P* < 0.0001	*P* = 0.0008	*P* = 0.0224
Psychological status	X38	No	0.765 ± 0.140	0.750 ± 0.120	0.778 ± 0.143	0.771 ± 0.158	0.768 ± 0.134
		Yes	0.806 ± 0.148	0.770 ± 0.125	0.806 ± 0.153	0.773 ± 0.143	0.781 ± 0.128
			*P* < 0.0001	*P* < 0.0001	*P* < 0.0001	*P* = 0.9048	*P* < 0.0001
EQ-5D scores	X39	No	0.813 ± 0.140	0.751 ± 0.132	0.781 ± 0.162	0.729 ± 0.131	0.747 ± 0.131
		Yes	0.750 ± 0.143	0.769 ± 0.109	0.802 ± 0.130	0.822 ± 0.157	0.804 ± 0.125
			*P* < 0.0001	*P* < 0.0001	*P* < 0.0001	*P* < 0.0001	*P* < 0.0001
Duration of treatment regimen (in months)	X41	No	0.709 ± 0.116	0.759 ± 0.098	0.786 ± 0.112	0.838 ± 0.172	0.806 ± 0.132
		Yes	0.790 ± 0.145	0.759 ± 0.124	0.791 ± 0.151	0.766 ± 0.148	0.771 ± 0.131
			*P* < 0.0001	*P* = 0.1224	*P* = 0.0054	*P* < 0.0001	*P* < 0.0001
Type of insulin used	X42	No	0.760 ± 0.138	0.755 ± 0.116	0.785 ± 0.138	0.785 ± 0.158	0.778 ± 0.132
		Yes	0.803 ± 0.147	0.762 ± 0.128	0.796 ± 0.156	0.762 ± 0.145	0.770 ± 0.131
			*P* < 0.0001	*P* < 0.0001	*P* < 0.0001	*P* < 0.0001	*P* = 0.0008
Use of insulin	X43	No	0.764 ± 0.141	0.750 ± 0.119	0.779 ± 0.143	0.776 ± 0.159	0.770 ± 0.134
		Yes	0.804 ± 0.146	0.769 ± 0.125	0.804 ± 0.153	0.768 ± 0.143	0.778 ± 0.129
			*P* < 0.0001	*P* < 0.0001	*P* < 0.0001	*P* < 0.0001	*P* = 0.0095
Times of insulin use	X44	No	0.774 ± 0.142	0.750 ± 0.121	0.779 ± 0.147	0.762 ± 0.153	0.763 ± 0.133
		Yes	0.788 ± 0.146	0.764 ± 0.123	0.797 ± 0.149	0.778 ± 0.150	0.780 ± 0.130
			*P* < 0.0001	*P* < 0.0001	*P* < 0.0001	*P* < 0.0001	*P* < 0.0001
Dose of basal insulin (U)	X45	No	0.751 ± 0.138	0.744 ± 0.118	0.771 ± 0.141	0.775 ± 0.164	0.766 ± 0.137
		Yes	0.812 ± 0.145	0.772 ± 0.125	0.808 ± 0.153	0.770 ± 0.139	0.781 ± 0.127
			*P* < 0.0001	*P* < 0.0001	*P* < 0.0001	*P* = 0.0001	*P* < 0.0001
Dose of non-basal insulin in morning (U)	X46	No	0.763 ± 0.138	0.746 ± 0.121	0.773 ± 0.144	0.763 ± 0.160	0.761 ± 0.136
		Yes	0.805 ± 0.149	0.774 ± 0.123	0.809 ± 0.151	0.782 ± 0.141	0.787 ± 0.125
			*P* < 0.0001	*P* < 0.0001	*P* < 0.0001	*P* < 0.0001	*P* < 0.0001
Dose of non-basal insulin in afternoon (U)	X48	No	0.776 ± 0.142	0.755 ± 0.120	0.785 ± 0.145	0.771 ± 0.153	0.771 ± 0.132
		Yes	0.795 ± 0.149	0.766 ± 0.125	0.800 ± 0.153	0.774 ± 0.149	0.779 ± 0.131
			*P* < 0.0001	*P* < 0.0001	*P* < 0.0001	*P* = 0.3904	*P* = 0.0016
Number of oral drugs	X49	No	0.731 ± 0.127	0.742 ± 0.115	0.770 ± 0.133	0.773 ± 0.187	0.765 ± 0.149
		Yes	0.791 ± 0.146	0.762 ± 0.123	0.794 ± 0.150	0.772 ± 0.145	0.775 ± 0.129
			*P* < 0.0001	*P* < 0.0001	*P* < 0.0001	*P* = 0.0158	*P* = 0.5867
Use of other types of drugs	X50	No	0.692 ± 0.084	0.692-0.692	0.738 ± 0.065	0.738-0.739	0.860 ± 0.036
		Yes	0.707 ± 0.084	0.707-0.707	0.716 ± 0.093	0.716-0.716	0.869 ± 0.048
			*P* < 0.0001	*P* < 0.0001	*P* < 0.0001	*P* < 0.0001	*P* < 0.0001
Use of metformin	X51	No	0.687 ± 0.098	0.715 ± 0.108	0.736 ± 0.120	0.782 ± 0.205	0.751 ± 0.156
		Yes	0.798 ± 0.145	0.766 ± 0.123	0.799 ± 0.150	0.771 ± 0.141	0.777 ± 0.127
			*P* < 0.0001	*P* < 0.0001	*P* < 0.0001	*P* < 0.0001	*P* = 0.0002
Dose of metformin	X52	No	0.767 ± 0.141	0.761 ± 0.118	0.792 ± 0.141	0.784 ± 0.159	0.781 ± 0.133
		Yes	0.798 ± 0.147	0.758 ± 0.126	0.790 ± 0.155	0.762 ± 0.144	0.767 ± 0.130
			*P* < 0.0001	*P* = 0.5662	*P* = 0.5470	*P* < 0.0001	*P* < 0.0001
Type of manufacturers of metformin	X53	No	0.759 ± 0.138	0.747 ± 0.119	0.775 ± 0.142	0.767 ± 0.162	0.764 ± 0.136
		Yes	0.805 ± 0.148	0.770 ± 0.124	0.805 ± 0.152	0.776 ± 0.141	0.782 ± 0.127
			*P* < 0.0001	*P* < 0.0001	*P* < 0.0001	*P* = 0.0377	*P* < 0.0001
α-Glucosidase inhibitors	X54	No	0.708 ± 0.120	0.739 ± 0.112	0.764 ± 0.128	0.799 ± 0.180	0.776 ± 0.143
		Yes	0.809 ± 0.144	0.766 ± 0.125	0.800 ± 0.154	0.763 ± 0.139	0.773 ± 0.128
			*P* < 0.0001	*P* < 0.0001	*P* < 0.0001	*P* < 0.0001	*P* = 0.0007
Sulfonylureas	X55	No	0.746 ± 0.135	0.741 ± 0.119	0.768 ± 0.140	0.769 ± 0.168	0.762 ± 0.139
		Yes	0.816 ± 0.145	0.775 ± 0.123	0.811 ± 0.152	0.775 ± 0.135	0.784 ± 0.123
			*P* < 0.0001	*P* < 0.0001	*P* < 0.0001	*P* = 0.8960	*P* < 0.0001
DPP-4 inhibitors	X57	No	0.761 ± 0.139	0.753 ± 0.117	0.783 ± 0.140	0.779 ± 0.159	0.774 ± 0.134
		Yes	0.804 ± 0.147	0.765 ± 0.127	0.798 ± 0.155	0.766 ± 0.143	0.774 ± 0.130
			*P* < 0.0001	*P* < 0.0001	*P* < 0.0001	*P* < 0.0001	*P* = 0.5225

### Sample size assessment

**Figure 5:**
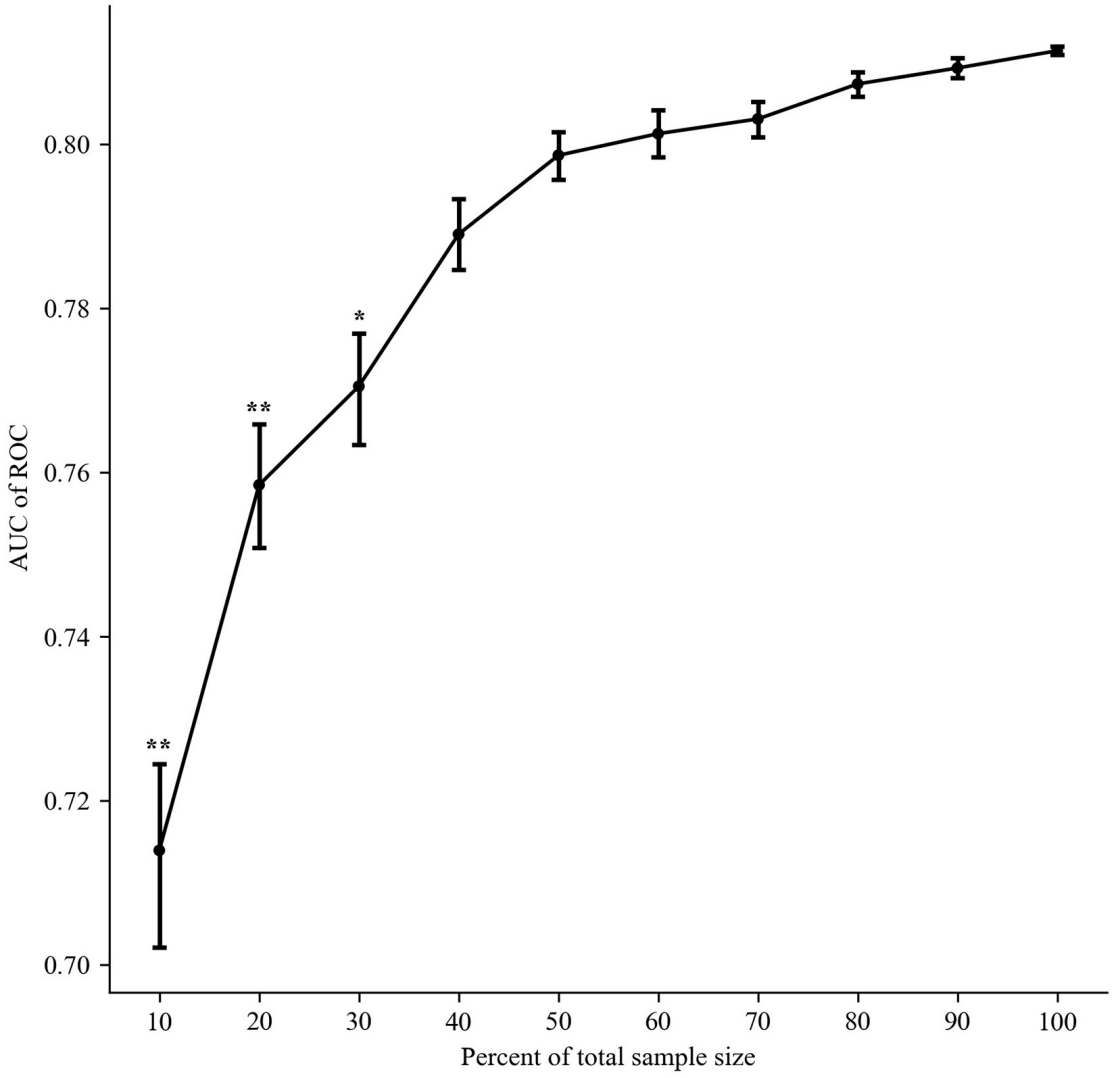
The impact of sample data size on model performances (mean ± SD).

As shown in Figure 5, with the size of sample data incorporated into the model from small to large, the values of AUC continued to increase. When the sample size was extremely small ( ≤ 30%), compared with the 100% sample size, the SDs of AUC were dispersed, and the AUCs were statistically significant (*P* < 0.05). As the sample size increased, the above situation was alleviated (*P*>0.05). In addition, the growth rate of AUC slowed down when the sample size was more than or equal to 40%. These results indicated that the performance of the proposed model might be affected less when expanding the sample size. The sample size was suitable for the prediction model construction.

## Discussion

Early detection of non-adherence to medication in patients with T2D will help devise strategies for personalized treatment. In this research, we developed a total of 1,080 models for the prediction of adherence in patients with T2D. The AUC, accuracy, precision, recall, F1 score, and AUPRC of the best model were 0.8369, 0.9474, 0.6792, 0.7912, and 0.9574, respectively. Meanwhile, various methods in model development and variables were validated by univariate analyses. Interestingly, the imputing method, the sampling method, the variable selection method, and the machine learning algorithm applied in the best model were the same as the results of univariate analysis. Additionally, variables with high importance scores in the best model were similar to the results of variable validation, which provided a positive contribution to the model prediction.

The adherence to the medication of patients with T2D has received great attention worldwide (24, 25). Nonadherence is associated with bad outcomes, including increased mortality and avoidable healthcare costs. Previous studies reported models to predict drug non-adherence in Crohn's disease maintenance therapy (26), patients with hypertension (27), and patients with heart failure (28). However, few studies reported on prediction models of non-adherence to medication in patients with T2D. Intelligence technology is becoming more prevalent in healthcare as a tool to improve practice patterns and patient outcomes (29–31). With technology development, ensemble models have been commonly used to explore disease progression in the field of molecular biology (32–36). Recently, the ensemble algorithm has been frequently applied to develop prediction models (37, 38). In our prior study, we reported that the ensemble algorithm was superior to the Bayesian network, KNN, SVM, C&R Tree, and CHAID (19). In this study, we added more machine learning classifications, including XGBoost, Bernoulli Naive Bayes, SGD, etc. Additionally, the ensemble algorithm was still the best.

Many variables have previously been reported to associate with drug adherence, such as age, population, level of education, etc. For example, according to the data from the National Health Insurance Service-National Sample Cohort (NHIS-NSC) of Korea, adherence consistently increased as the age increased until 69 years and started to decrease from the age of 70 years. When the same number of drugs was taken, the proportion of adherent patients according to age featured an inverted U-shape with a peak at 60–69 years (39). Additionally, Aditama et al. (25) stated that the factors influencing non-adherence included complex instructions for taking medication, the absence of a reminder, the unwanted side effects of the drug, feeling of repetition, feeling that the drugs were ineffective, and concern for the effects of the drug on the kidney. Therefore, more patient-related and drug-related variables were considered in this study, including the number of comorbid diseases, EQ-5D scores, number of oral drugs, use of other types of drugs, and so on.

The results of the univariate analysis suggested that more variables can improve the accuracy of the prediction model (AUC = 0.063, *P* < 0.001). In clinical research, more variables mean collecting more data and increasing the missing data. Thus, feature selection plays an important role in the field of machine learning. In this study, no screening (marked as Not), Boruta, and LassoCV were performed. Boruta is a feature selection algorithm to identify the minimal set of relevant variables, which was applied in the best model. According to the variable importance score, the ten most important variables were age, present FBG values, present HbA1c values, present RBG values, BMI, duration of the treatment regimen, interval of measurement, waistline, weight, and course of diabetes. Glycemic control in patients with T2D can be accessed *via* the following three key parameters: glycated hemoglobin (HbA1c), FBG, and RBG. The results of variable importance demonstrated that patients with non-adherence should strongly encourage to monitor their blood glucose and receive reinforced education.

## Limitation

First, this was a single-center study, and the patient profile might be biased and not representative of the Chinese as a whole. People from Sichuan Province may have different distributions of risk factors than patients in other areas of China. A large multicenter sample study is desired, which can verify the applicability of the model. However, for some variables, recall bias still exists, such as the intensity of exercise and exercise sessions.

## Conclusion

In summary, the present research introduced 1,080 machine-learning models to predict non-adherence in patients with T2D and proposed an ensemble model with better classifier performance. This study also reconfirmed that variables including age, BMI, and interval of measurement were risk factors for non-adherence. We are in the process of developing a mobile App or a web server for caregivers and patients in an effort to integrate the adherence enhancement intervention into daily T2D management.

## Data availability statement

The original contributions presented in the study are included in the article/supplementary material, further inquiries can be directed to the corresponding authors.

## Ethics statement

The studies involving human participants were reviewed and approved by the Ethics Committee of the Sichuan Provincial People's Hospital (Approval # 2018-53). The patients/participants provided their written informed consent to participate in this study.

## Author contributions

ML and XL contributed to data analysis and writing and approval of the final manuscript. HY and RY assisted in the face-to-face questionnaire. RT and YY were responsible for designing and coordinating the research. XW was involved in the questionnaire design, data analysis, model design, and contributed to revision of the manuscript. All authors agree to be accountable for the content of this study.

## Funding

This study was funded by the National Natural Science Foundation of China (Grant No. 72004020), the Key Research and Development Program of Science and Technology Department of Sichuan Province (Grant No. 2019YFS0514), the Postgraduate Research and Teaching Reform Project of the University of Electronic Science and Technology of China (Grant No. JYJG201919), the Research Subject of Health Commission of Sichuan Province (Grant No. 19PJ262), Sichuan Science and Technology Program (Grant No. 2021YJ0427), and Scientific Research Foundation of Sichuan Provincial People's Hospital (Grant No. 2022BH10).

## Conflict of interest

The authors declare that the research was conducted in the absence of any commercial or financial relationships that could be construed as a potential conflict of interest.

## Publisher's note

All claims expressed in this article are solely those of the authors and do not necessarily represent those of their affiliated organizations, or those of the publisher, the editors and the reviewers. Any product that may be evaluated in this article, or claim that may be made by its manufacturer, is not guaranteed or endorsed by the publisher.
